# Disease and economic burden for rare diseases in Taiwan: A longitudinal study using Taiwan’s National Health Insurance Research Database

**DOI:** 10.1371/journal.pone.0204206

**Published:** 2018-09-21

**Authors:** Jason C. Hsu, Huai-Chueh Wu, Wen-Chia Feng, Chih-Ho Chou, Edward Chia-Cheng Lai, Christine Y. Lu

**Affiliations:** 1 School of Pharmacy and Institute of Clinical Pharmacy and Pharmaceutical Sciences, College of Medicine, National Cheng Kung University, Taiwan; 2 School of Medicine, College of Medicine, National Cheng Kung University, Taiwan; 3 National Health Insurance Administration, Ministry of Health and Welfare, Taiwan; 4 Department of Neurology, Chi Mei Hospital, Tainan, Taiwan; 5 Department of Population Medicine, Harvard Medical School and Harvard Pilgrim Health Care Institute, MA, United States of America; National Yang-Ming University Hospital, TAIWAN

## Abstract

**Background:**

High-cost orphan drugs are becoming increasingly available to treat rare diseases that affect a relatively small population. Little attention has been given to the prevalence of rare diseases and their health-related economic burden in Taiwan.

**Objectives:**

This study examined the national trends in the prevalence of rare diseases and their health-related economic burden (including medication costs) in Taiwan.

**Methods:**

Rare disease-related claims data from 2003–2014 (12 years) from the National Health Insurance Research Database were used in this study. We used a time series analysis to assess trends in the yearly rates of treated patients with rare diseases, overall healthcare use, and expenditures, including drugs.

**Results:**

During the 12-year study period, the estimated prevalence of rare diseases increased from 10.57 to 33.21 per 100,000 population, an average rate of a 19.46% increase per year. Total health expenditures for treatment of rare diseases increased from US$18.65 million to US$137.44 million between 2003 and 2014, accounting for 0.68% of the total national health expenditures in 2014. Drug expenditures for treatment of rare diseases increased from US$13.24 million to US$121.98 million between 2003 and 2014, which accounted for 71.00% and 88.75% of the health expenditures for patients with rare diseases in 2003 and 2014, respectively. In 2014, we found a 20.43-fold difference in average health expenditures and a 69.46-fold difference in average drug expenditures between patients with rare diseases and the overall population.

**Conclusions:**

The prevalence of rare diseases and the related economic burden have grown substantially in Taiwan over the past 12 years, and these trends are likely to continue. Drug expenditures accounted for almost 90% of health expenditures for rare diseases. Further analyses are underway to examine the economic burden of individual rare diseases.

## Introduction

The ice bucket challenge that has spread globally since 2014 is an activity involving dumping a bucket of ice water on a person’s head to promote awareness of amyotrophic lateral sclerosis, a rare disease, to encourage donations to research. Due to this, rare disease-related associations around the world have received a large number of donations, most of which were invested in the research of rare diseases. Following the ice bucket challenge, rare disease-related issues have received more attention, and policies have been implemented in many countries to improve the treatment of rare diseases.[1]

Most countries define rare diseases based on disease prevalence, but the criteria vary from country to country. For instance, in the United States and Japan, a rare disease is one with a prevalence fewer than 200,000 persons and 50,000, respectively [2, 3]. The EU defines rare diseases as fewer than 5 per 10,000 persons. [3–5] Compared to the criteria in other countries, the general definition of a rare disease in Taiwan (< 1/10,000 persons) [2] is stricter. In the United States, nearly 7,000 rare diseases have been identified [6], and there are around 6,000–8,000 rare diseases in the EU. [7, 8] However, only 330 and 220 rare diseases have been defined in Japan and in Taiwan [9], respectively.

With advances in medical science and technology for rare disease treatment as well as the rising awareness of the importance of patients’ human rights and issues of availability and the cost of orphan drugs have gained significant attention. However, health and drug utilization and the economic impacts of rare diseases are largely unknown in many countries, including Taiwan. The objectives of this study were to examine 2003–2014 longitudinal trends in the prevalence and expenditure of rare diseases in Taiwan. We also analyzed these trends for two specific rare diseases—amyotrophic lateral sclerosis (ALS) and multiple sclerosis (MS)–because ALS is the main targeted rare disease in the ice bucket challenge activity, and MS is another rare disease with similar symptoms to those of ALS.

## Method

### Data sources

Nationwide claims data from Taiwan’s National Health Insurance Research Database (NHIRD) were used in this study. The database contains information from a nationwide, mandatory-enrollment, single-payer healthcare system created in 1995. Nearly 99% of the Taiwanese population (around 23 million residents) is enrolled, and this system contracts with 97% of the hospitals and clinics throughout the country. The National Health Insurance (NHI) covers a wide range of prescription medicines as well as inpatient and outpatient medical services. All yearly claims data related to patients with rare diseases, including details of prescription and insurer spending between 2003 and 2014 (12 years) were retrieved from Taiwan’s National Health Insurance Research Database. Using the registry for the catastrophic illness patient database, patients with rare diseases were identified according to the International Classification of Diseases, 9^th^ edition (ICD-9) diagnosis codes for all identifiable rare diseases in Taiwan, for example, ICD-9 codes for ALS and MS are “335.2” and “340” respectively. [10] In addition, yearly population data were obtained from the Department of Household Registration, Taiwan Ministry of Interior.[11] All analyses were carried out with SAS software, Version 9.4 (SAS Institute, Cary, NC).

### Measurements

To examine trends in the prevalence of rare diseases, use of health services and drugs for rare diseases, and their expenditures, we calculated the yearly number of services and prescriptions and treatment costs in Taiwan from 2003 to 2014. The prevalence was estimated by using the number of patients with rare diseases divided by the number of all people in the same year. We also calculated the number of patients with rare diseases by age and gender in 2014 to represent the distribution of cases. The treatment costs included health (overall treatment) and drug expenditures (cost of all prescription drugs, including orphan drugs).

Then, we calculated the percentage of the use and costs of health and drug expenditures out of the total use and total costs for the entire population. The percentage of health expenditures (including drug expenditures) on rare diseases was estimated by using health expenditures for patients with rare diseases divided by the health expenditure for all people; the percentage of drug expenditure for rare disease treatments out of total drug expenditure was estimated by using drug expenditures for rare disease treatment divided by total drug expenditures for all people.

Furthermore, the number of patients with rare diseases was used as the denominator to adjust yearly reimbursed expenditures. The average health expenditures among patients with rare diseases was estimated as the health expenditures of patients with rare diseases divided by the number of patients with rare diseases; the average drug expenditures among patients with rare diseases was estimated by dividing the drug expenditures of patients with rare diseases by the number of patients with rare diseases.

The 2014 exchange rate and yearly health care consumer price index were obtained and calculated from the historical exchange rate database of the Bank of Taiwan and the Taiwan National Statistics database. We anchored the cost at 2014 and adjusted the other years using the health care consumer price index (HCCPI) between 2002 and 2014. Then, we used the exchange rate (30.368) for 2014 to convert New Taiwan dollars to US dollars.

## Results

### Trends in the prevalence of rare diseases in Taiwan

The number of patients with rare diseases gradually increased from 2,390 in 2003 to 7,785 in 2014 (the growth rate between 2003 and 2014 was 225.73%, which was 20.52% per year) (Table 1). The prevalence of rare diseases steadily increased from 10.57 per 100,000 people in 2003 to 33.21 in 2014 (growth rate: 19.46% per year). The number of patients with ALS also increased from 112 in 2003 to 478 in 2014 (growth rate: 29.71% per year), and its prevalence rose from 0.5 in 2003 to 2.04 in 2014 (growth rate: 28.32% per year). The number of patients with MS increased from 406 in 2003 to 1,101 in 2014 (growth rate: 15.56% per year), and its prevalence rose from 1.8 in 2003 to 4.7 in 2014 (growth rate: 14.68% per year).

**Table 1:** Prevalence of rare diseases in Taiwan by year.

Year	2003	2004	2005	2006	2007	2008	2009	2010	2011	2012	2013	2014	Growth rate (2003–2014)	Growth rate per year
**Number of Patients with rare diseases**	**2,390**	**3,034**	**3,706**	**4,286**	**4,873**	**5,371**	**5,889**	**6,210**	**6,600**	**7,059**	**7,352**	**7,785**	**225.73%**	**20.52%**
**Number of people covered by NHI**	**22,604,550**	**22,689,122**	**22,770,383**	**22,876,527**	**22,958,360**	**23,037,031**	**23,119,772**	**23,162,123**	**23,224,912**	**23,315,822**	**23,373,517**	**23,443,753**	**3.71%**	**0.34%**
**Prevalence of rare diseases (per 100,000 people)**	**10.57**	**13.37**	**16.28**	**18.74**	**21.23**	**23.31**	**25.47**	**26.81**	**28.42**	**30.28**	**31.45**	**33.21**	**214.07%**	**19.46%**
**Number of Patients with Amyotrophic lateral sclerosis (ALS)**	**112**	**171**	**211**	**229**	**276**	**299**	**329**	**365**	**385**	**419**	**448**	**478**	**326.79%**	**29.71%**
**Prevalence of ALS (per 100,000 people)**	**0.50**	**0.75**	**0.93**	**1.00**	**1.20**	**1.30**	**1.42**	**1.58**	**1.66**	**1.80**	**1.92**	**2.04**	**311.51%**	**28.32%**
**Number of Patients with Multiple sclerosis (MS)**	**406**	**489**	**552**	**615**	**678**	**767**	**834**	**884**	**964**	**1,041**	**1,052**	**1,101**	**171.18%**	**15.56%**
**Prevalence of MS (per 100,000 people)**	**1.80**	**2.16**	**2.42**	**2.69**	**2.95**	**3.33**	**3.61**	**3.82**	**4.15**	**4.46**	**4.50**	**4.70**	**161.47%**	**14.68%**

Prevalence of all rare diseases (per 100,000 people) = number of patients with all rare diseases / number of people in the same year * 100,000

Growth rate = (the value in 2014 –the value in 2003) / the value in 2003.

**Fig 1:**
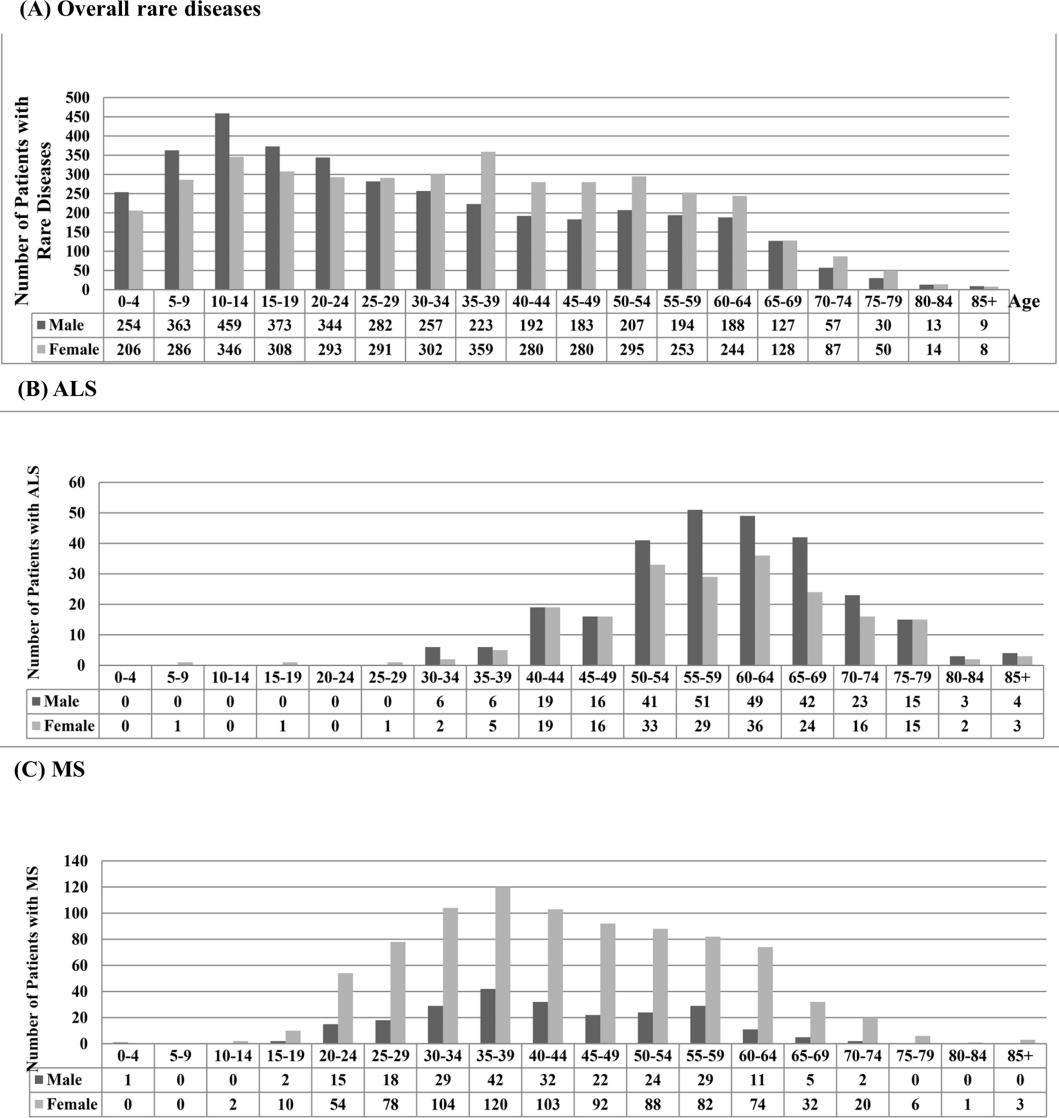
Number of patients with rare diseases by age and gender in Taiwan in 2014: (A) Overall rare diseases, (B) ALS, (C) MS.

Fig 1(A) shows the prevalence of rare diseases by age and gender in Taiwan in 2014. A higher prevalence was found in males as compared to females among those younger than 24 years old, but a lower prevalence was found in males than in females among those older than 25 years of age. The prevalence was highest for 10–14 year-old males and 5–9 year-old females. Among patients with rare diseases, patients aged less than 20 accounted for one-third of all patients in 2014. Most ALS patients were 50–70 years old, and the number of male patients in all age groups was slightly higher than that of females. Most MS patients were 30–50 years-old, and the number of female patients in all age groups was significantly higher than that of males (Fig 1(B) and 1(C)).

### Trends in health expenditures for rare diseases in Taiwan

Table 2 presents the trend in health expenditures for rare diseases in Taiwan. Health expenditures for patients with rare diseases grew sharply from US$18,647,411 (accounting for 0.13% of health expenditures for all people) in 2003 to US$137,444,523 (accounting for 0.68% of health expenditures for all people) in 2014. The growth rate between 2003 and 2014 was 637.07%, which was 57.92% per year. The health expenditures of patients with ALS also rapidly grew from US$1,199,745 (accounting for 0.01% of the total national health expenditures) in 2003 to US$4,379,012 (accounting for 0.02% of the total national health expenditures) in 2014. The growth rate between 2003 and 2014 was 265%; 24.09%). The health expenditures of patients with MS sharply grew from US$2,679,099 (accounting for 0.02% of the total national health expenditures) in 2003 to US$9,104,389 (accounting for 0.04% of the total national health expenditures) in 2014. The growth rate between 2003 and 2014 was 239.83% (2003–2014) and was 21.80% per year.

**Table 2:** Health expenditures for treatment of rare diseases in Taiwan by year.

Year	2003	2004	2005	2006	2007	2008	2009	2010	2011	2012	2013	2014	Growth rate (2003–2014)	Growth rate per year
**Health care consumer price index**	**81.10%**	**82.72%**	**86.07%**	**88.95%**	**92.49%**	**94.49%**	**95.05%**	**95.67%**	**97.42%**	**98.26%**	**99.42%**	**100.00%**		
**Health expenditures of patients with rare diseases (US$)**	**18,647,411**	**25,022,090**	**30,538,076**	**41,061,531**	**52,490,093**	**62,453,840**	**68,774,372**	**76,291,738**	**87,478,826**	**106,616,627**	**119,588,293**	**137,444,523**	**637.07%**	**57.92%**
**Health expenditures of patients with ALS (US$)**	**1,199,745**	**1,886,128**	**2,319,270**	**2,817,172**	**3,386,289**	**4,004,365**	**3,988,212**	**3,972,107**	**4,352,494**	**4,377,158**	**4,244,775**	**4,379,012**	**265.00%**	**24.09%**
**Health expenditures of patients with MS (US$)**	**2,679,099**	**3,638,180**	**4,114,818**	**4,671,306**	**5,488,452**	**6,153,418**	**6,861,778**	**7,131,273**	**7,109,819**	**7,378,284**	**8,669,961**	**9,104,389**	**239.83%**	**21.80%**
**Health expenditure for all population (US$)**	**14,344,492,864**	**16,212,896,662**	**16,065,671,400**	**15,676,444,176**	**15,674,923,107**	**16,152,201,583**	**16,893,391,882**	**17,223,601,420**	**17,777,165,566**	**18,953,515,939**	**19,518,365,385**	**20,260,126,481**	**41.24%**	**3.75%**
**Rate of health expenditures for patients with rare diseases for the total population**	**0.13%**	**0.15%**	**0.19%**	**0.26%**	**0.33%**	**0.39%**	**0.41%**	**0.44%**	**0.49%**	**0.56%**	**0.61%**	**0.68%**	**0.55%**	**0.05%**
**Rate of health expenditures for patients with ALS among all rare diseases**	**6.43%**	**7.54%**	**7.59%**	**6.86%**	**6.45%**	**6.41%**	**5.80%**	**5.21%**	**4.98%**	**4.11%**	**3.55%**	**3.19%**	**-3.25%**	**-0.30%**
**Rate of health expenditures for patients with MS among all rare diseases**	**14.37%**	**14.54%**	**13.47%**	**11.38%**	**10.46%**	**9.85%**	**9.98%**	**9.35%**	**8.13%**	**6.92%**	**7.25%**	**6.62%**	**-7.74%**	**-0.70%**
**Average health expenditures for patients with rare diseases (US$)**	**7,802**	**8,247**	**8,240**	**9,580**	**10,772**	**11,628**	**11,678**	**12,285**	**13,254**	**15,104**	**16,266**	**17,655**	**126.28%**	**11.48%**
**Average health expenditures for patients with ALS (US$)**	**10,712**	**11,030**	**10,992**	**12,302**	**12,269**	**13,393**	**12,122**	**10,882**	**11,305**	**10,447**	**9,475**	**9,161**	**-14.48%**	**-1.32%**
**Average health expenditures for patients with MS (US$)**	**6,599**	**7,440**	**7,454**	**7,596**	**8,095**	**8,023**	**8,228**	**8,067**	**7,375**	**7,088**	**8,241**	**8,269**	**25.31%**	**2.30%**
**Average health expenditures for all patients (US$)**	**635**	**715**	**706**	**685**	**683**	**701**	**731**	**744**	**765**	**813**	**835**	**864**	**36.18%**	**3.29%**

The 2014 exchange rate and yearly health care consumer price index were obtained and calculated from the historical exchange rate database of the Bank of Taiwan and the Taiwan National Statistics database. Percentage of health expenditures on rare disease = health expenditures for patients with rare diseases / health expenditures for all people x100%. Average health expenditures = health expenditures for patients with rare diseases / number of patients with rare diseases. Growth rate = (the value in 2014 –the value in 2003) / the value in 2003.

**Fig 2:**
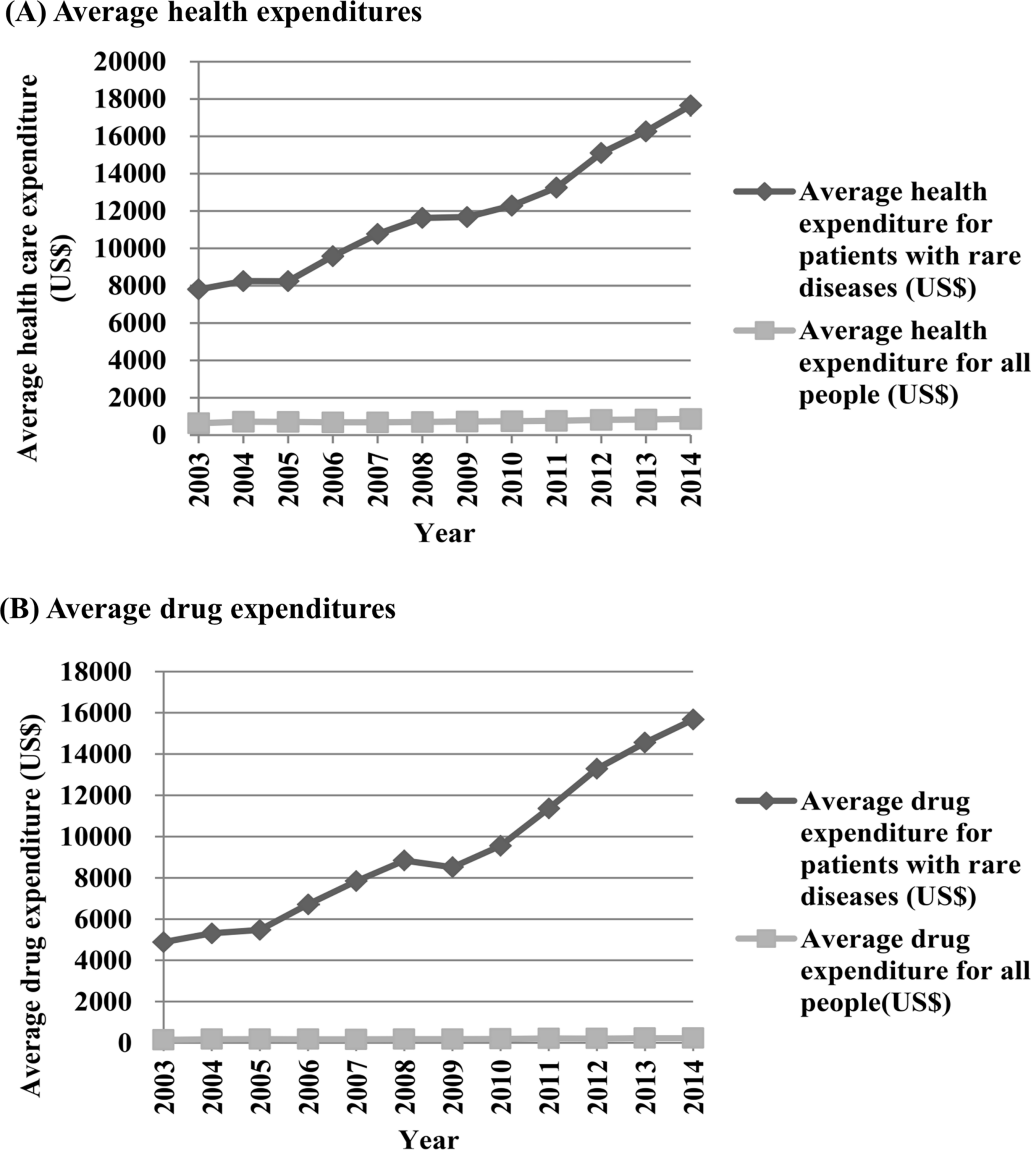
**Average health and drug expenditures among patients with rare diseases and among the total population (2003–2014): (A) Average health expenditures, (B) Average drug expenditures.** * Average health expenditures for patients with rare diseases = health expenditures for patients with rare diseases / number of patients with rare diseases * Average health expenditures for all people = health expenditures for all patients / number of all people * Average drug expenditures for patients with rare diseases = drug expenditures for patients with rare diseases / number of patients with rare diseases * Average drug expenditures for all people = drug expenditures for all diseases / number of all people.

There was a gradual increase in average health expenditures for patients with rare diseases from 2003 (US$7,802) to 2014 (US$17,655), for which the growth rate between 2003 and 2014 was 126.28% (2003–2014), which was 11.48% per year (Fig 2(A)). The average health expenditures for patients with ALS decreased from US$10,712 to US$9,161 with a -1.32% growth rate per year; the average health expenditures for patients with MS increased from US$6.599 to US$8,269 with a 2.30% growth rate per year. In comparison, the average health expenditures for all people steadily increased from US$635 to US$864 (the growth rate between 2003 and 2014 was 36.18% (2003–2014), which was 3.29% per year).

### Trends in drug expenditure of rare diseases in Taiwan

Table 3 presents the trend in drug expenditures for rare diseases in Taiwan. Drug expenditures for patients with rare diseases grew sharply from US$13,239,156 (accounting for 71.00% of health expenditures for all patients with rare diseases and 0.35% of drug expenditures for the total population) in 2003 to US$137,444,523 (accounting for 88.75% of health expenditures for all people and 2.31% of drug expenditures for the total population) in 2014. The growth rate of drug expenditures for patients with rare diseases between 2003 and 2014 was 74.67% per year). On the other hand, non-drug expenditure for patients with rare diseases also rose from US$5,408,254 to US$15,463,453 (growth rate: 16.90% per year).

**Table 3:** Drug expenditures for treatment of rare diseases in Taiwan by year.

Year	2003	2004	2005	2006	2007	2008	2009	2010	2011	2012	2013	2014	Growth rate (2003–2014)	Growth rate per year
**Health care consumer price index**	**81.10%**	**82.72%**	**86.07%**	**88.95%**	**92.49%**	**94.49%**	**95.05%**	**95.67%**	**97.42%**	**98.26%**	**99.42%**	**100.00%**	**23.30%**	**2.12%**
**Drug expenditures of patients with rare diseases (US$)**	**13,239,156**	**17,728,400**	**21,490,642**	**30,826,224**	**41,291,203**	**49,242,372**	**54,551,336**	**61,844,751**	**72,732,035**	**91,460,269**	**104,875,592**	**121,981,070**	**821.37%**	**74.67%**
**Drug expenditures of patients with ALS (US$)**	**336,540**	**553,734**	**661,182**	**795,388**	**850,819**	**899,993**	**997,516**	**839,185**	**832,627**	**744,209**	**798,876**	**867,896**	**157.89%**	**14.35%**
**Drug expenditures of patients with MS (US$)**	**453,371**	**2,381,840**	**2,812,673**	**3,291,900**	**4,108,836**	**4,839,317**	**5,317,957**	**5,561,079**	**5,449,904**	**5,684,813**	**7,034,468**	**7,663,989**	**1590.44%**	**144.59%**
**Drug expenditures for the total population (US$)**	**3,836,987,013**	**4,355,131,213**	**4,288,859,524**	**4,223,865,345**	**4,165,617,578**	**4,307,589,150**	**4,507,168,086**	**4,515,656,382**	**4,810,028,649**	**4,755,609,595**	**5,100,943,049**	**5,288,461,538**	**37.83%**	**3.44%**
**Non-drug expenditures of patients with rare diseases (US$)**	**5,408,254**	**7,293,690**	**9,047,434**	**10,235,307**	**11,198,891**	**13,211,468**	**14,223,036**	**14,446,987**	**14,746,790**	**15,156,358**	**14,712,701**	**15,463,453**	**185.92%**	**16.90%**
**Non-drug expenditures of patients with ALS (US$)**	**863,205**	**1,332,394**	**1,658,087**	**2,021,784**	**2,535,469**	**3,104,372**	**2,990,696**	**3,132,922**	**3,519,867**	**3,632,949**	**3,445,899**	**3,511,117**	**306.75%**	**27.89%**
**Non-drug expenditures of patients with MS (US$)**	**2,225,728**	**1,256,340**	**1,302,145**	**1,379,406**	**1,379,616**	**1,314,101**	**1,543,821**	**1,570,194**	**1,659,915**	**1,693,471**	**1,635,493**	**1,440,400**	**-35.28%**	**-3.21%**
**Non-drug expenditures for the total population (US$)**	**10,507,505,851**	**11,857,765,450**	**11,776,811,876**	**11,452,578,831**	**11,509,305,528**	**11,844,612,433**	**12,386,223,796**	**12,707,945,038**	**12,967,136,918**	**14,197,906,345**	**14,417,422,336**	**14,971,664,943**	**42.49%**	**3.86%**
**Rate of drug expenditures divided by health expenditures for all patients with rare diseases**	**71.00%**	**70.85%**	**70.37%**	**75.07%**	**78.66%**	**78.85%**	**79.32%**	**81.06%**	**83.14%**	**85.78%**	**87.70%**	**88.75%**	**17.75%**	**1.61%**
**Rate of drug expenditure divided by health expenditures for all patients with ALS**	**28.05%**	**29.36%**	**28.51%**	**28.23%**	**25.13%**	**22.48%**	**25.01%**	**21.13%**	**19.13%**	**17.00%**	**18.82%**	**19.82%**	**-8.23%**	**-0.75%**
**Rate of drug expenditures divided by health expenditures for all patients with MS**	**16.92%**	**65.47%**	**68.35%**	**70.47%**	**74.86%**	**78.64%**	**77.50%**	**77.98%**	**76.65%**	**77.05%**	**81.14%**	**84.18%**	**67.26%**	**6.11%**
**Rate of drug expenditures for patients with rare diseases among the total population**	**0.35%**	**0.41%**	**0.50%**	**0.73%**	**0.99%**	**1.14%**	**1.21%**	**1.37%**	**1.51%**	**1.92%**	**2.06%**	**2.31%**	**1.96%**	**0.18%**
**Rate of drug expenditures for patients with ALS among all population**	**0.01%**	**0.01%**	**0.02%**	**0.02%**	**0.02%**	**0.02%**	**0.02%**	**0.02%**	**0.02%**	**0.02%**	**0.02%**	**0.02%**	**0.01%**	**0.00%**
**Rate of drug expenditures for patients with MS among the total population**	**0.01%**	**0.05%**	**0.07%**	**0.08%**	**0.10%**	**0.11%**	**0.12%**	**0.12%**	**0.11%**	**0.12%**	**0.14%**	**0.14%**	**0.13%**	**0.01%**
**Average drug expenditures for patients with rare diseases (US$)**	**5,539**	**5,843**	**5,799**	**7,192**	**8,473**	**9,168**	**9,263**	**9,959**	**11,020**	**12,957**	**14,265**	**15,669**	**182.86%**	**16.62%**
**Average drug expenditures for patients with ALS (US$)**	**3,005**	**3,238**	**3,134**	**3,473**	**3,083**	**3,010**	**3,032**	**2,299**	**2,163**	**1,776**	**1,783**	**1,816**	**-39.57%**	**-3.60%**
**Average drug expenditures for patients with MS (US$)**	**1,117**	**4,871**	**5,095**	**5,353**	**6,060**	**6,309**	**6,376**	**6,291**	**5,653**	**5,461**	**6,687**	**6,961**	**523.36%**	**47.58%**
**Average drug expenditures for all patients (US$)**	**170**	**192**	**188**	**185**	**181**	**187**	**195**	**195**	**207**	**204**	**218**	**226**	**32.89%**	**2.99%**

The 2014 exchange rate and yearly health care consumer price index were obtained and calculated from the historical exchange rate database of the Bank of Taiwan and the Taiwan National Statistics database. Percentage of drug expenditures over health expenditures on rare disease = drug expenditures for all patients with rare diseases / health expenditures for all patients with rare diseases; Percentage of drug expenditures for rare diseases treatments among total drug expenditures = drug expenditures for rare disease treatment / total drug expenditures for all people. Average drug expenditures = drug expenditures of patients with rare diseases / number of patients with rare diseases. Growth rate = (the value in 2014 –the value in 2003) / the value in 2003.

The drug expenditures of patients with ALS grew from US$336,540 (accounting for 28.05% of health expenditures for all patients with ALS) in 2003 to US$867,896 (accounting for 19.82% of health expenditures for all patients with ALS) in 2014. The growth rate of the drug expenditures of patients with ALS between 2003 and 2014 was 14.35% per year). The drug expenditures of patients with MS sharply grew from US$453,371 (accounting for 16.92% of health expenditures for all patients with MS) in 2003 to US$7,663,989 (accounting for 81.14% of health expenditures for all patients with MS) in 2014. The growth rate of the drug expenditures of patients with MS between 2003 and 2014 was 144.59% per year). The growth rates of the non-drug expenditures of patients with ALS and MS were 27.89% and -3.21% per year, respectively.

There was a gradual increase in the average drug expenditures of patients with rare diseases from 2003 (US$5,539) to 2014 (US$15,669), and the growth rate between 2003 and 2014 was 182.86% (2003–2014), which was 16.62% per year (Fig 2(B)). The average drug expenditures of patients with ALS decreased from US$3,005 to US$1,816 with a -3.60% growth rate per year; the average drug expenditures of patients with MS increased from US$1,117 to US$6,961, with a 47.58% growth rate per year. The average drug expenditures for the total population slightly increased from US$170 to US$226 (the growth rate between 2003 and 2014 was 32.89% (2003–2014), which was 2.99% per year).

## Discussion

Between 2003 and 2014, the prevalence of rare diseases increased 3.14-fold (ALS: 4.12 fold; MS: 2.61 fold) although the prevalence rates were still very low in 2014 (rare diseases: 33.21; ALS: 2.04; MS: 4.70 per 100,000 people). The increase in the prevalence of rare diseases might be related to growing awareness of rare diseases and better diagnosis. For many decades, patients, families, and physicians have had a limited awareness of rare diseases. Clinicians might have had little experience with patients suffering from rare diseases due to their low prevalence, and symptoms are often hidden by more common illnesses and initially may appear to be of only minor concern.[12] However, in recent years, rare disease-related issues have received more attention, and policies have been implemented in many countries to improve the treatment of rare diseases.[1] For instance, increased and more advanced disease screening help identify certain rare diseases early in life; family history and genetic screening help with earlier diagnosis, and genetic testing confirms disease diagnoses.[13, 14]

This study found a sharp increase in the national economic burden possibly due to the growth in the number of diagnosed patients and increasing availability of orphan drugs over time.[14, 15] The expenditures for health care for rare diseases and orphan drugs have increased in many countries as well as in Taiwan over the last decade. Globally, drug expenditures for orphan drugs accounted for 13.7% of total drug expenditures in 2013, and they have been estimated to reach 15.9% in 2018.[16] In the US, expenditures for orphan drugs totaled around US$30 billion (8.9% of total pharmaceutical expenditures) in 2013.[17] The expenditures for orphan drugs in the EU accounted for 3.3% of the total pharmaceutical expenditures in 2010, and expenditures were predicted to reach 4.6% in 2016.[18] In Taiwan, the drug expenditures for treatment of rare diseases were estimated to have been around 2.31% of total pharmaceutical expenditures in 2014, which was lower than the estimates in the US and EU, possibly due to differences in rare disease definitions (fewer diseases were identified as rare diseases in Taiwan) and differences in the costs of health services and drugs.

Compared with the average health and drug expenditures for the Taiwanese population, expenditures for patients with rare diseases have been very high, probably because of the high prices of orphan drugs. For example, the average drug expenditure of a patient with ALS was US$1,816 per year in 2014, which is 8 times higher the average drug expenditure for the total population (US$226) in Taiwan. The high price of orphan drugs is not surprising. Given the rarity of each disease, the high costs of research and development during clinical trials and the smaller markets for orphan drugs lead manufacturers to seek much higher drug prices to ensure profits.[1, 19, 20]

The high costs of orphan drugs are huge economic burden for the NHI; the issue of drug affordability for treatment of rare diseases in Taiwan requires the attention of policy makers since the majority of orphan drugs for rare diseases are covered and reimbursed by the National Health Insurance. To gain access to unmarketed orphan drugs in Taiwan, requests can be directed to the government to import orphan drugs.[21] Those who have one of the following circumstances can apply for rewards: supply, manufacture, research, and development of orphan drugs, the introduction of rare disease drugs, including orphan drugs in the prescription set, and project applications for rare disease drugs.[22] In relation to patient affordability, patients can apply for financial support for orphan drugs, diagnosis, life-sustaining special nutritional food, and home health care equipment. Furthermore, the government organizes educational and public awareness activities related to rare diseases with assistance from schools, civic groups, and mass media. When rare disease patients encounter problems in school enrollment, employment, or life support, the government coordinates with relevant organizations to provide assistance.[23]

To date, Taiwan's “Rare Diseases and Drugs Review Committee” has recognized 220 rare diseases [9], 162 kinds of rare drugs [24], and 40 special rare nutritious foods [25]. In response to increasing health care and drug costs for rare diseases, many related regulations and policies have been proposed or amended. To provide more medical subsidies, in addition to continuing to strengthen screening services, the government also revised the “The Rare Disease and Orphan Drug Act” in 2010. In addition to continuing to provide special nutritional products for patients with rare diseases and expanding the subsidies for international inspections, the expenditures for low- and middle-income patients will be fully subsidized, and the expenses for home medical care equipment rental, domestic confirmation diagnosis, nutrition consultation, emergency medical treatment for patients with rare diseases are also covered. In 2011, the cost of special nutrition needed to sustain life became fully subsidized. These policies will reduce the economic burden for patients with rare diseases.

Before the establishment of the Rare Diseases Foundation, patients with rare diseases in Taiwan faced difficulties in obtaining medical resources. The prevention and control work could not keep up with the times and technology, and the medical system and the social system were almost completely unable to care for these rare disease patients. The Taiwan Rare Disease Foundation was established in June 1999, and it has been promoting medical human rights, patient rights, eugenics, and the reform of policies. In order to assist families with rare diseases facing economic difficulties, various types of financial subsidies have been provided for many years to help patients with significant medical expenses, expensive medical equipment, sudden injury to their home economic pillars, or life difficulties caused by illness. The foundation established the “Medical Relief Fund” in 1999. Its main purpose is to subsidize the genetic testing of rare diseases as well as to assist in the diagnosis of patients, surgery, drugs, and other related medical expenses. In addition, the foundation also established the “Life Relief Fund” in 2000 to help patients cope with the difficult conditions in their lives and ensure the stability and development of their patients. Furthermore, the “Rare Diseases Relief Fund” was established in 2005 and was based on the principle of subsidizing social security, social assistance, or other welfare systems at all levels of government that fail to pay or pay only the relevant annuity care costs.

There are some limitations to this study. First, this study was aimed toward an examination of the current trends of prevalence and related expenditures in the treatment of rare diseases in Taiwan. We focused on the national economic burden (health and drug expenditures) related to overall rare diseases instead of individual patients; thus, we did not analyze the trend in patients’ out-of-pocket costs or clinical outcomes associated with orphan drug treatments. Second, this study did not estimate the prevalence and related expenditures for specific rare diseases. However, we took two iconic rare diseases—ALS and MS—as examples for in-depth analyses. Further studies are valuable to identify the specific rare diseases with the highest prevalence for 10–14 year-old males and 5–9 year-old females, as well as the specific rare diseases with the highest growth rate of total health/drug expenditures and the average of health/drug expenditures. Finally, future studies are needed to explore the associations between various factors (including patient characteristics, disease severity, comorbidity, and physician knowledge and behavior) as well as the national/individual economic burden for rare diseases. Research is also needed to examine the impacts of various interventions, such as the launch of each new medication, changes in clinical guidelines or insurance reimbursement policies, on the overall health and drug expenditures for rare diseases.

## Conclusion

In recent years, the economic burden of rare diseases has become an important issue in many countries, including Taiwan. We conducted a well-designed observational study using national healthcare data to characterize the current landscape of rare disease prevalence and health and drug expenditures in Taiwan for the last decade. We found increases in the prevalence of rare diseases and sharp increases in national health and drug expenditures over time. Drug expenditures contributed to 88.75% of health expenditures for rare diseases in 2014. With more new, costly drugs in the development pipeline, drug expenditures are likely to increase further. The findings of this study provide an empirical basis for future studies on rare diseases and orphan drugs.
